# Mixed method evaluation of a learning from excellence programme for community health workers in Neno, Malawi

**DOI:** 10.1186/s12913-024-10686-w

**Published:** 2024-03-19

**Authors:** Maartje Kletter, Bronwyn Harris, Emilia Connolly, Chifundo Namathanga, Basimenye Nhlema, Henry Makungwa, Benson Chabwera, Benson Phiri, Celia Brown

**Affiliations:** 1 The University of Manchester, Oxford Road, Manchester, M13 9PL UK; 2 Warwick Medical School, University of Warwick, Coventry, CV4 7AL UK; 3 Partners In Health/Abwenzi Pa Za Umoyo, Neno, PO Box 56, Malawi

**Keywords:** Community Health workers, Positive psychology, Mixed method, Logic model, Evaluation

## Abstract

**Background:**

Community Health Workers (CHWs) play an essential role in linking communities to facility-based healthcare. However, CHW programmes have often been hampered by low levels of staff motivation, and new tools aimed at improving staff motivation and work environment are needed. One such intervention is the “Learning from Excellence” (LfE) programme. We aimed to assess feasibility, outputs, and impact of a co-designed LfE programme on CHW motivation, in Neno District.

**Methods:**

We conducted a convergent mixed-method evaluation of the LfE programme. Co-design of the programme and forms took place between October 2019 and January 2020. LfE forms submitted between September and November 2020 were analysed using descriptive statistics and memos summarising answers to the open-ended question. To investigate experiences with LfE we conducted in-depth semi-structured interviews with key stakeholders, CHWs, and site supervisors, which were analysed thematically. A pre-post intervention questionnaire was developed to assess the impact of the co-designed LfE intervention on CHW motivation and perceived supervision. Outcomes were triangulated into a logic model.

**Results:**

In total 555 LfE forms were submitted, with 34.4% of CHWs in Neno District submitting at least one LfE report. Four themes were identified in the interviews: LfE implementation processes, experience, consequences, and recommendations. A total of 50 CHWs participated in the questionnaire in January 2020 and 46 of them completed the questionnaire in December 2020. No statistically significant differences were identified between pre-and post-LfE measurements for both motivation (Site F: *p* = 0.86; Site G: *p* = 0.31) and perceived supervision (Site F: *p* = 0.95; Site G: *p* = 0.45). A logic model, explaining how the LfE programme could impact CHWs was developed.

**Conclusions:**

Many CHWs participated in the LfE intervention between September 2020 and November 2020. LfE was welcomed by CHWs and stakeholders as it allowed them to appreciate excellent work in absence of other opportunities to do so. However, no statistically significant differences in CHW motivation and perceived supervision were identified. While the intervention was feasible in Neno District, we identified several barriers and facilitators for implementation. We developed a logic model to explain contextual factors, and mechanisms that could lead to LfE outcomes for CHWs in Neno District. The developed logic model can be used by those designing and implementing interventions like LfE for health workers.

**Supplementary Information:**

The online version contains supplementary material available at 10.1186/s12913-024-10686-w.

## Background

Community Health Workers (CHWs) play a critical role in linking communities to healthcare by providing promotional, preventive, screening, and sometimes curative services, especially in settings with a critical shortage of human resources for health [1]. CHWs are defined by the World Health Organization as: *“health workers based in communities who are either paid or volunteer, who are not professionals, and who have fewer than two years training but at least some training”* [2]. CHW programmes vary widely in programmatic structure and scope. Due to their proximity to communities, skills, and lack of formally trained staff CHWs can be overloaded [3]. This can lead to performance variation and low levels of staff motivation when programmes are brought to scale [4–11].

Studies on performance and motivation in CHWs have shown that monetary compensation, whether as a salary or stipend, is valued highly by CHWs and allows them to dedicate themselves to the job, regardless of whether they are a professional cadre [6, 12–15]. However, financial incentives alone are insufficient for unlimited health worker motivation [16]. Previous studies have shown that in addition to financial incentives, non-financial incentives have helped CHWs sustain motivation to perform their assigned tasks timely and with high quality [6, 12, 17]. Examples of non-financial incentives include appreciation by trained healthcare staff, and community appreciation for the work CHWs do, which can lead to a feeling of pride among CHWs as they support their communities [6, 12, 17]. However, non-financial incentives do not ensure high performance and cannot compensate for the lack of financial incentives and resources [12].

To enhance performance and programmatic efficacy, CHW programmes are examining new motivation tools and improving their staff’s work environment. One new motivational intervention is the Learning from Excellence programme (LfE), which was introduced in the United Kingdom (UK) in 2014. LfE’s aim is to capture excellence in healthcare, to study excellence, and to improve resilience and staff morale and create new opportunities for learning. LfE was introduced to redress the balance as there is a preoccupation in healthcare with avoiding error and harm [18]. LfE focuses on the strengths of people and aims to identify what makes individuals and teams in healthcare settings flourish. It is a programme based on positive psychology (PP), defined as “the scientific study of human strengths and virtues” by Martin Seligman [19, 20].

LfE could potentially increase CHW motivation and performance, as identified in a recent study among CHWs in a rural setting in Malawi, where they represent a critical element of the community health system [21].

The LfE intervention currently consists of a written form that can be used to report on fellow colleagues’ excellence, and feedback about who has been reported for excellence and why. There is no definition for what excellence entails, and anything can be reported if the reporter considers the reported event an act of excellence. In 2018 an exploratory study regarding the impact of LfE on health professionals in the United Kingdom (UK) was conducted. Participants expressed how LfE improved team morale, positive emotions, motivation, resilience, and relationships among team members [22]. However, the impact of interventions like LfE on lay health workers out of the facility setting, like CHWs, is unknown, with potential impact in connecting these normally solitary workers.

To explore the impact of LfE for CHWs in rural Malawi, we aimed to develop a co-designed LfE form and assess the feasibility, suitability, and outputs of the developed LfE form, and to evaluate the co-designed LfE reporting form based on these outputs. This paper also evaluates the impact of a co-designed LfE programme on CHW motivation, including job satisfaction and perceived supervision. To explain how the co-designed LfE intervention impacts CHW performance we adapted a logic model, as designed in earlier studies [23, 24]. The logic model was adapted with the help of realist informed methodology: we identified context-mechanism-outcome (CMO) configurations explaining how interventions like LfE could potentially impact CHWs in Neno District. The outcomes of this study, including the logic model, can be used by others designing and implementing LfE interventions for health workers, particularly in community-based rural settings.

## Methods

We conducted a convergent mixed method evaluation [25] of the co-designed LfE programme for CHWs in Neno District. A mixed method approach was chosen to not only investigate the usage of LfE forms and the impact on motivation and perceived supervision, but to also investigate CHW’s experiences with LfE and barriers or facilitators they experienced during implementation of the intervention. Quantitative data consisted of completed LfE forms from all catchment areas in Neno district as well as questionnaire data collected from CHWs in two catchment areas. Additionally individual in-depth interviews were conducted with stakeholders in the CHW programme, Site Supervisors and CHWs. We chose a convergent mixed method model, where quantitative and qualitative data are collected and analysed separately due to time constraints [25]. We chose. Mixed method methodology as combining qualitative and quantitative data provided us with a more comprehensive overview of the impact of CHW and its implementation than only one type of data would have done. After separate analyses we converged the data into a logic model, explaining how LfE could impact CHWs in Neno District.

### Setting

This study was conducted in Neno District in collaboration with Partners in Health (PIH), an international not-for-profit, non-governmental organisation and the local Ministry of Health in Malawi. Neno District is a remote, rural district in Southwest Malawi, home to an estimated 150,000 people in 2018, with limited accessibility due to very few tarmac roads and mountainous terrain [26]. Within the District, there are 14 catchment areas, each served by a health facility– two hospitals and 12 primary facilities. However, over 60% of the population still reports difficulty accessing the facilities due to distance [27, 28].

The CHW programme was first implemented in 2007 by PIH and the Ministry of Health to improve patient adherence, psychosocial support, and clinical outcomes in tuberculosis (TB) and HIV clients in the Neno District [29, 30]. However, to respond to the disease burden within the district and community feedback, in 2017 the CHW programme was adjusted to a polyvalent model where a CHW was assigned to a household regardless of disease. The programme expanded to focus on eight major public health areas: TB, HIV and other sexually transmitted infections, non-communicable diseases, family planning, maternal and neonatal health, child health and malnutrition screening in under five-year-olds [31].

The programme is a three-tiered model with household-level CHWs, senior CHWs, and site supervisor roles that provide support to the facility-based community health care worker– the Health Surveillance Assistant (HSA) [32]. The primary roles of household-level CHWs are to visit households monthly, provide health education and promotion activities, screen for disease, and refer household members to the health facility for diagnostic and treatment services. They provide psychosocial support, treatment monitoring, and appointment reminders when disease is identified. Senior CHWs maintain the CHW role with fewer households assigned and supervise CHWs along with community TB sputum collection points. Both cadres of CHWs work as volunteers but receive a monthly stipend to compensate for time and transport. Site supervisors are based at the health facility and provide support to all CHWs in their catchment area. They have a direct line of communication with the senior CHWs.

Additional information about the design of the LfE programme and implementation is provided in supplementary file 1.

### Quantitative data - questionnaire

#### Questionnaire design

Quantitative data were collected through a self-designed questionnaire consisting of ten questions, designed in collaboration with CHW leadership in Neno District. The starting point for questionnaire development was the logic model explaining the impact of LfE on health personnel in the UK, as developed in an exploratory study on the effect of LfE on National Health Service (NHS) Hospital trusts in the UK [22], and the outcomes of our systematic review (181).

CHW leadership and MK identified the following outcomes for CHWs in the Neno District: (i) motivation, including general motivation, organisational commitment, and job satisfaction; and (ii) perceived supervision, which was similar to supportive mentorship. Motivation was chosen as it had been previously identified as a potential outcome of LfE or interventions like LfE [22, 24]. Perceived supervision was selected due to the previously identified ‘breaking down of hierarchies’, ‘feeling of community and ‘high-quality relationships’ [22, 24], and the expectation that LfE could lead to supportive mentorship. We chose not to include more than ten questions to keep the questionnaire short, to encourage the busy CHWs to fully participate.

An overview of the selected questions, including the construct they intend to measure, is provided in Table 1, which also includes adaptations to questions aimed at better matching the context of CHWs in Neno District. Items were scored using a 5-point Likert scale from strongly disagree [1] to strongly agree [5]. The questionnaire was translated into Chichewa and back translated into English to check for validity.

**Table 1:** Process of development of CHW questions for the evaluation questionnaire

	Question	Construct	Adapted	Reason for adaptation
1 [33]	In general, I am very satisfied with my job	Motivation (general motivation)	NA	
2 [34]	I am actively seeking other employment	Motivation (job satisfaction)	If I could, I would do a different job	CHWs have limited education and there are few opportunities for them so we changed it to find out if they would potentially change roles if they could
3 [33]	This health facility really inspires me to do my very best on the job	Motivation (organisational commitment)	I feel my work is appreciated and valued by the SCHW and Site Supervisor	More specific, which was thought to make it easier for CHWs to answer the question
4 [33]	I am proud to be working for this facility	Motivation (organisational commitment)	I am proud to be a CHW	CHWs don’t work in a facility, so we focused on their role as CHW
5 [33]	I am not satisfied with my colleagues at work	Motivation (job satisfaction)	I feel part of a Community Health Team	PIH preferred the question to be phrased positively as they expected a more honest response, additionally, we made it more specific
6 [35]	My supervisor meets with me regularly to discuss problems and solutions	Perceived supervision	I feel able to discuss work-related problem with other CHWs and SCHWs	More specific, which was thought to make it easier for CHWs to answer the question
7 [33]	Feel motivated to work hard	Motivation (general motivation)	I feel motivated to work as hard as I can	More specific, which was thought to make it easier for CHWs to answer the question
8 [33]	Only do this job to get paid	Motivation (general motivation)	In only do this job to get paid at the end of the month	More specific, which was thought to make it easier for CHWs to answer the question
9 [35]	My supervisor meets with me regularly	Perceived supervision	The SCHW meets with me regularly	More specific was thought to make it easier for CHWs to answer the question
10 [35]	My supervisor helps me to update my knowledge	Perceived supervision	The SCHW helps me to update my knowledge and skills	More specific was thought to make it easier for CHWs to answer the question. Additionally, not just knowledge but also skills included

CHW = Community Health Worker

NA = Not Applicable

PIH = Partners in Health

SCHW = Senior Community Health Worker

#### Questionnaire participants

At each site, 25 CHWs were included in the study, based on a crude power calculation with a power of 0.8, alpha of 0.05 and a medium effect size of 0.5 for the outcome change. Using paired data, this calculation returned a minimum of 34 participants to complete the pre-and post-intervention questionnaire. As we expected some drop-out between pre-and post-intervention data collection, we included 50 CHWs in the baseline questionnaire. Participants were selected by the site supervisor, who invited them during the monthly facility-based meeting (where CHWs receive their stipend and submit data collected during their work as CHWs). Participants were approached for participation by the CHW programme manager and Site Supervisors who selected participants based on age, gender, and time working as CHWs.

#### Quantitative data collection

LfE was first implemented in one catchment area in July 2020 and rolled out in the entire district in August 2020. We initially planned to implement the co-designed intervention in two of the 14 catchment areas because CHWs in these areas use a mobile health (mHealth) application to conduct their work. However, due to more urgent programming needs with the COVID-19 pandemic, it was impossible to integrate the LfE intervention into the mHealth application. Instead, we implemented the LfE intervention in the entire district as a paper-based intervention.

Pre-intervention data were collected before the COVID-19 pandemic, when the intervention was focused on the two catchment areas (Site F and Site G), on the monthly date CHWs visited the facility for stipends and data collection in January 2020. The informed consent form was read aloud to CHWs, and they were encouraged to ask questions. Once informed consent was obtained, the questionnaire was read aloud, and CHWs were given some time to look through the questionnaire themselves and ask any questions for clarification.

As we only had pre-intervention data from Site F and Site G, and the intention was to assess changes over time, post-intervention quantitative data were only collected at these sites. The post-implementation questionnaire was administered in November 2020 at Site F and Site G, including the CHWs who participated in the pre-intervention questionnaire. Collected data were anonymised and added to an excel spreadsheet. Due to miscommunication, most CHWs in Site F did not write their names on the post-intervention questionnaire, which meant paired data analysis was not possible.

#### Quantitative data analysis

On completion of data collection, pre- and post-intervention data analysis was conducted in January 2021. Outcomes for question 2 and question 8, the negatively phrased questions were converted so that all questions were scored between 1 (lowest) and 5 (highest). The internal validity and consistency of the questionnaire were checked with the help of inter-question correlations and Cronbach’s alpha. Descriptive statistics were obtained, and we calculated the median, interpolated median and interquartile ranges for each question, assigning numerical values to each response on the Likert scale from strongly disagree = 1 to strongly agree = 5. Additionally, we calculated the median and interquartile ranges for the constructs assessed by more than one question: motivation and perceived supervision. We conducted a paired data analysis by construct, comparing the baseline questionnaire with the post-intervention questionnaire using a Wilcoxon Signed Rank test for the paired data from Site G, while a Mann Whitney U test for the unpaired data from Site F was conducted. Statistical significance was set at *p* < 0.05. Stata (v17) was used for data analysis.

### Qualitative data - in-depth interviews

#### Interview participants

We interviewed participants from different levels within the programme including PIH CHW leadership stakeholders, site supervisors and CHWs. Four stakeholders from CHW leadership at PIH were selected for participation in the interviews: the Community Health Director (CHD), CHW Programme Manager and two CHW Programme Officers. These stakeholders were selected as they had been involved in the roll-out and implementation of the LfE intervention, as well as for their leadership and continuous involvement in the CHW programme.

We purposively selected seven *site supervisors* to participate. Site supervisors were chosen if their site had a large number of CHWs participating, high quality of reports, low number of reports, low percentage of CHWs participating, CHWs submitting multiple reports, a high percentage of CHWs reporting each other, and because the site did not stand out in any way. Fifteen CHWs, five from each site, were selected from three different sites: Site F, Site G, and Site K. These were selected due to their variation in participation in the LfE intervention and Site F and Site G participated in the quantitative study as well. Site G additionally showed many CHWs filling in multiple reports, while in Site F a small percentage of CHWs participated. Site K was chosen as the CHW team considered the quality of the reports to be very high.

To learn more about CHWs’ experiences with participation, being reported for excellence, or non-participation in LfE, we purposively selected five CHWs per site as follows: two CHWs who filled in a report, two CHWs who were reported for excellence and one CHW who did not participate in the LfE project. All CHWs were randomly selected through an Excel Random Number generator.

#### Qualitative data collection

Interviews were conducted between the 25th of January 2021 and the 5th of February 2021. CN conducted all interviews with CHWs and Site Supervisors, as these were conducted in Chichewa. Except for one interview, the interviews conducted by CN were conducted in person and, with the consent of the interviewee, recorded and transcribed. One interview was conducted over the phone. This interview could not be recorded, and CN took detailed notes. All face-to-face interviews were conducted in a quiet place at the health facility. MK performed the three interviews with stakeholders via a virtual platform. These interviews were held in English and recorded with the permission of the participants.

During the interviews, the following topics were discussed: LfE design, LfE implementation, the impact of LfE, including contractual factors, mechanisms and outcomes that play a role, and the future of the LfE. All recorded interviews were transcribed verbatim and translated by CN if held in Chichewa.

#### Qualitative data analysis

To check MK’s understanding of the interviews conducted in Chichewa, memos were developed to summarise the Site Supervisor and CHW interviews. The memos were shared and discussed with CN.

A thematic analysis was conducted by MK [36], who familiarised themselves with the data, through developing memos of translated interviews and by transcribing and reading through the interviews conducted in English [36]. The interviews were subsequently coded in nVivo by MK. BH also coded three randomly selected interviews to ensure consistency. BH and MK discussed the findings in a meeting.

Once coding was completed, similar codes were grouped to form sub-themes based on similarity [36]. The sub-themes were subsequently grouped, again based on similarity, to develop themes. A memo was written about each theme to summarise the findings.

### Data synthesis

The quantitative and qualitative data were triangulated into a logic model, explaining how LfE can impact CHWs in Neno District. We adapted a logic model developed in a previous study [24] and assessed if the contextual factors, mechanisms, and outcomes of this evaluation converged with the developed logic model or if they diverged, in which case the logic model was adapted accordingly. The initial logic model is presented in supplementary file 2.

We categorised contextual factors as follows, as per previous studies [37, 38]: factors before design and implementation of the intervention (factors present in the organisation that support enthusiasm for interventions), factors during the design (factors that support the uptake of the intervention) and factors during the intervention itself (factors that support the effectiveness of the intervention). The logic model was adapted to include newly identified factors.

## Results

Between September 2020 and November 2020, a total of 555 LfE forms were submitted at 13 catchment areas. However, 34 reports did not include the role of the reporter. In total 390 (34.4% of CHWs in Neno District) CHWs submitted 521 reports (Table 2). At sites A, D, E, I, and L, CHWs only submitted one report each, while at all other sites (except site N) CHWs submitted multiple reports over the course of four months. Most reports per CHW were submitted at site G, where 61 CHWs submitted 131 reports (supplementary file 1).

### Questionnaires

The questionnaire consisted of 10 questions, but we decided not to include question 2 and question 8, the negatively phrased questions, in the statistical evaluation due to low correlations with the motivational construct both pre- and post-implementation and an increase in Cronbach’s alpha if questions were removed (see supplementary file 3).

The median, interquartile range and interpolated median scores per question before and after the implementation of LfE, as well as Spearman correlations between the different questions of the questionnaire before and after LfE, respectively are presented in supplementary file 3. Questionnaire outcomes pre-and post-intervention for motivation and perceived supervision are presented in Table 3. CHWs’ scores for the positively phrased.

**Table 2:** Overview reports per site

Site	Number of CHWs participating	Number of reports
A	14	14
B	9	13
C	33	62
D	31	31
E	43	43
F	25	30
G	61	131
H	11	12
I	6	6
J	33	49
K	29	34
L	41	41
M	54	55
Total	**390**	**521**

No statistically significant differences were identified between pre-and post-LfE measurements for neither motivation (*p* = 0.86 and *p* = 0.31 for Site F and Site G, respectively) nor perceived supervision (*p* = 0.95 and *p* = 0.45 for Site F and Site G, respectively).

### In-depth interviews

In total, 24 interviews were conducted: 14 interviews with CHWs, seven interviews with site supervisors, and three interviews with CHW leadership stakeholders. One CHW declined to participate, and one stakeholder could not do so due to a lack of time. We identified four themes: LfE implementation processes, LfE experiences, LfE consequences and recommendations.

**Table 3:** Overview of questionnaire outcomes

			Site F		Site G	
				N		N
Before	Motivation	Median	24	23	22	25
		IQR	22 to 25		21 to 24	
	Supervision	Median	15	23	14	25
		IQR	14 to 15		14 to 15	
After	Motivation	Median	24	19	23	18
		IQR	23 to 25		21 to 25	
	Supervision	Median	14.5	22	14	23
		IQR	14 to 15		13 to 15	
Change	Motivation	Median			1	18
		IQR			0 to 2	
	Supervision	Median			0	23
		IQR			-1 to 1	
Wilcoxon Signed Rank for change	Motivation				*p* = 0.31 (z = -1.01)	18
	Supervision				*p* = 0.45 (z = 0.75)	23
Mann-Whitney U Test for change	Motivation		*p* = 0.86 (z = -0.18)	42		
	Supervision		*p* = 0.95 (z = 0.063)	45		

IQR = Interquartile range

#### LfE implementation processes

Various facilitators for the uptake and outcomes of LfE were mentioned. CHWs said that the main reasons they decided to participate were encouragement by Site supervisors to participate in LfE. Additionally, support from, and good liaison with the CHW team from the DHO, as well as the step-by-step explanation of the LfE form, facilitated uptake.

*“If the forms were just handed over to us without any encouragement [from Site Supervisor], it could not have shown any impact.”– CHW 2*.

Time to reflect on their work and their colleague’s facilitated participation of CHWs. Reflection allowed CHWs to take a step back and look at their own performance and the performance of fellow CHWs, which helped them identify and report excellence.

*“[…] because for you to actually recognise or identify an area of excellence you must be in a space where you are reflecting on how you do your work how you can better the delivery of that work.”– Stakeholder 1*.

There seems to have been some miscommunication around implementation, as one CHW mentioned that they did not participate in the LfE programme because they were told to only report acts of excellence from the previous month. Another CHW mentioned that excellence had to be notified as soon as forms were handed out, and they did not have enough time to reflect on the performance of their fellow CHWs.

*“The time I wanted to write the report in that month [site Supervisor] said we should not vote excellence event from the previous month, so this made me to fail to write a report because the CHW whom I wanted to report on had done the excellent event the previous month.”– CHW 9*.

Other reported factors preventing participation included high workloads of CHWs and the heavy reliance on very busy Site supervisors during the implementation of the LfE intervention.

Lack of understanding led to CHWs not participating or CHWs reporting on various acts of excellence on one form, which was not what we intended. However, while many participating CHWs mentioned it took them a while to understand the LfE intervention, at the time the interviews were held, CHWs understood it well.

*“[…] the weaknesses were there since it came to us as a new thing and we couldn’t understand it better, but after being briefed now and again, we understood it better.”– CHW 6*.

#### LfE experience

CHWs, stakeholders and site supervisors mentioned the opportunity to appreciate CHWs through the LfE intervention. Before LfE was implemented, CHWs had not considered or expected praise as part of their working experience. However, LfE made CHWs realise that their work is worthy of appreciation.

*“This all because at first, we thought that our organisation can’t have such programme of appreciating each other and just thought that our work is not worth to be appreciated, but this programme brought a different idea in us.”– CHW 5*.

CHWs reported that the love of their work, positive emotions, and motivation felt when fellow CHWs are doing excellent work led to participation in the LfE intervention. When CHWs submitted a LfE report, it reminded them of the great work they, and their colleagues were doing, which encouraged them to participate again in the future.

*“[…] the hardworking spirit of colleagues facilitated me to fill the report and vote for them.”– CHW 11*.

However, on the other hand CHWs were worried that if they reported a colleague’s excellence, but were not reported themselves, it would look like the others were doing a better job and one CHW suggested that jealousy among CHWs increased due to the implementation of LfE.

*“I also think that there are some colleagues of mine maybe who saw those successes in others, but they failed to vote for them because of jealousy.”– CHW 7*.

Jealousy was often mentioned by CHWs and site supervisors as a reason for non-participation in the intervention. One CHW said that if they reported a colleague for excellence, it was expected that this colleague would report them for excellence as well; however due to jealousy among them, this did not always happen.

Site Supervisors also identified jealousy among CHWs as one of the barriers to participation in the intervention and lack of submitted LfE reports.

*“Some of the CHWs were failing to vote for others based on their reasons like family issues back in the villages where they stay […] if I can vote for such and such person then I will miss a reward in a way that their family will benefit more than mine.”– Site Supervisor 3*.

#### LfE consequence

The most identified consequence of the LfE intervention was that it encouraged CHWs to work hard. CHWs mentioned that LfE encouraged them to work hard as they wanted to be appreciated for their hard work and be in the spotlight.

*“[…] made people to be encouraged to vote for each other and also to work hard so that they should be in the spotlight.”– CHW 3*.

Particularly CHWs who did not work hard in the past are believed, by CHW leadership, site supervisors and CHWs themselves to have started working harder since the implementation of LfE because when CHWs took part in the LfE intervention, they wanted to be like those they were reporting for excellence. When CHWs realised they were doing a great job, or when they reported excellence, motivation to work hard increased. Site supervisors also mentioned that CHWs were eager to excel, making them work extra hard.

*“[…] the programme has brought an element of boosting self-esteem and a spirit of hardworking on those who have been voted.”– CHW 4*.

LfE was believed to lead to improved knowledge about acts of excellence. CHWs mentioned that the LfE form informed them about potential acts of excellence and what PIH considered excellence. The CHWs could subsequently emulate these excellent events in their work. Stakeholders reported that CHWs learnt from identifying and reporting on the excellence of their colleagues.

*“[form] was also giving us an idea that it’s a very good thing that if there is someone who had stopped taking medication and we are going there for encouragements then we want good health for them.”– CHW 10*.

Stakeholders’ knowledge of CHW work also increased because LfE provided insight into what was happening on the ground and allowed them to assess CHW performance and highlight areas of excellence. Stakeholders mainly gained insight regarding informal support networks, where CHWs ask their colleagues to support them.

*“I think [LfE] gives us an opportunity and a platform for people to learn from each other’s experiences.”– Stakeholder 1*.

Site supervisors mentioned that CHWs showed more interest in what their colleagues do since LfE was implemented, and they realised that it is important to highlight the good work of CHWs. Stakeholders mentioned potential outcomes could transcend the recognition platform as stakeholders hoped that over time, motivation, morale, and performance could improve due to the LfE intervention.

Additionally, LfE led to some unmet needs as they mentioned they expected rewards as a thank you for excellent performance. CHWs expected to be rewarded for their acts of excellence, which may explain CHWs reporting each other simultaneously (i.e. CHW A reporting CHW B and the other way around). The lack of rewards was thought to demotivate CHWs from participating in the intervention, as they did not see the benefit of doing so. This was influenced by a lack of feedback or failed interventions for CHWs in the past, which made them sceptical about new interventions.

*“This may be because the expectations which the CHWs had that if someone excels then they will receive a reward wasn’t there. When they realized this, it made them to lose interest in the whole programme and starting to see the programme to be of no value.”– Site Supervisor 7*.

#### Recommendations

One Site supervisor worried that the emphasis on excellence would demotivate CHWs, as not everyone excels. They recommended that the LfE programme be more generalised instead of focusing on acts of excellence only. Site supervisors also mentioned that the same CHWs were often reported for acts of excellence, and they wanted to restrict CHWs from voting for their friends.

*“The way the programme was introduced showed that only those who could do better are the ones to be appreciated, which demotivated others.”– Site Supervisor 1*.

One stakeholder recommended the presence of a dedicated LfE-person on the ground, as they can help encourage CHWs to participate, and follow up on forms and feedback results. One stakeholder aimed to fully integrate LfE into the CHW programme and other PIH programmes.

*“So, integrating [LfE] into the existing forums, so whether it’s at CHW level at senior level, really making it part of our day-to-day narrative and part of the discussions that we have […]”– Stakeholder 1*.

### Data synthesis: logic model

**Fig. 1:**
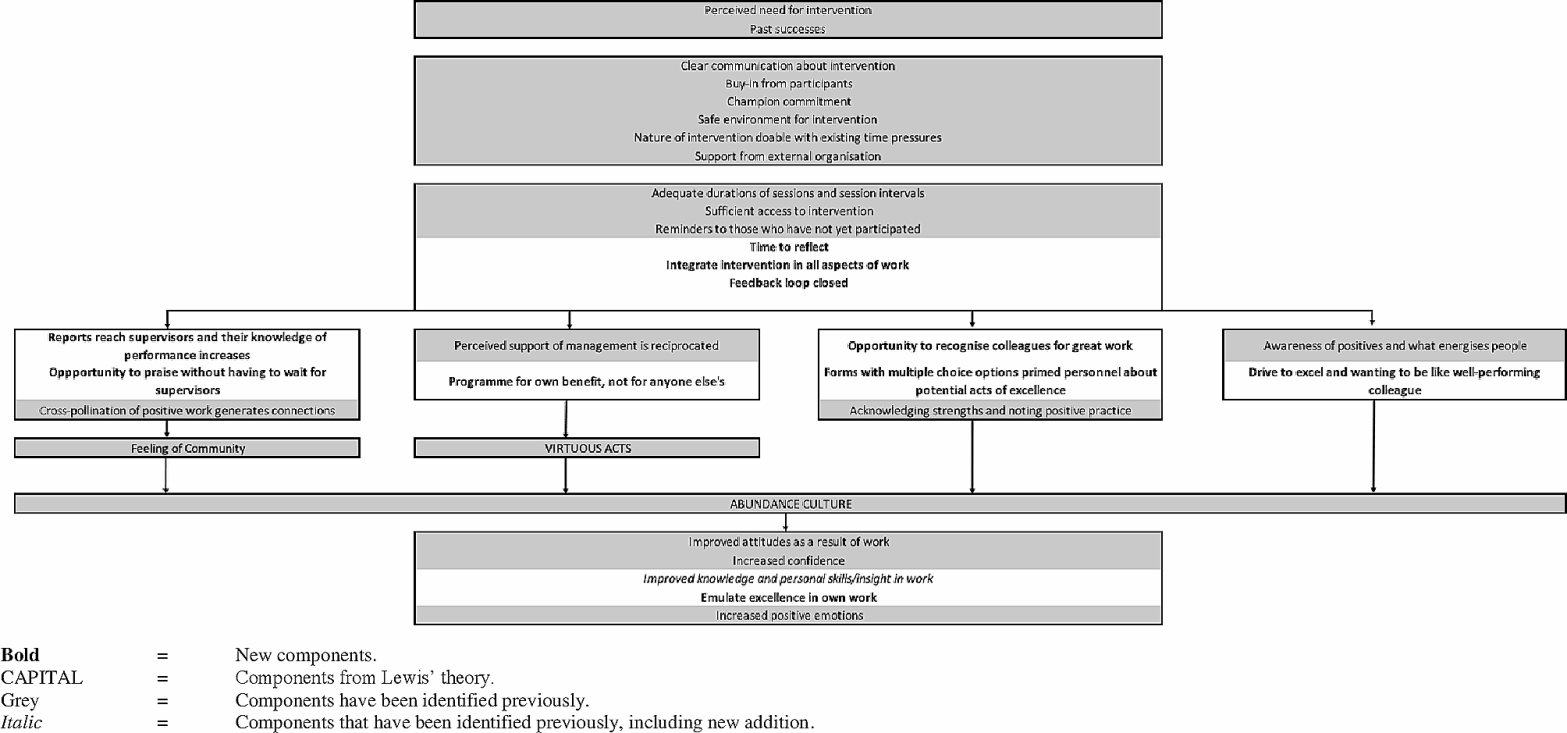
Logic model of factors identified in the mixed method study

**Fig. 2:**
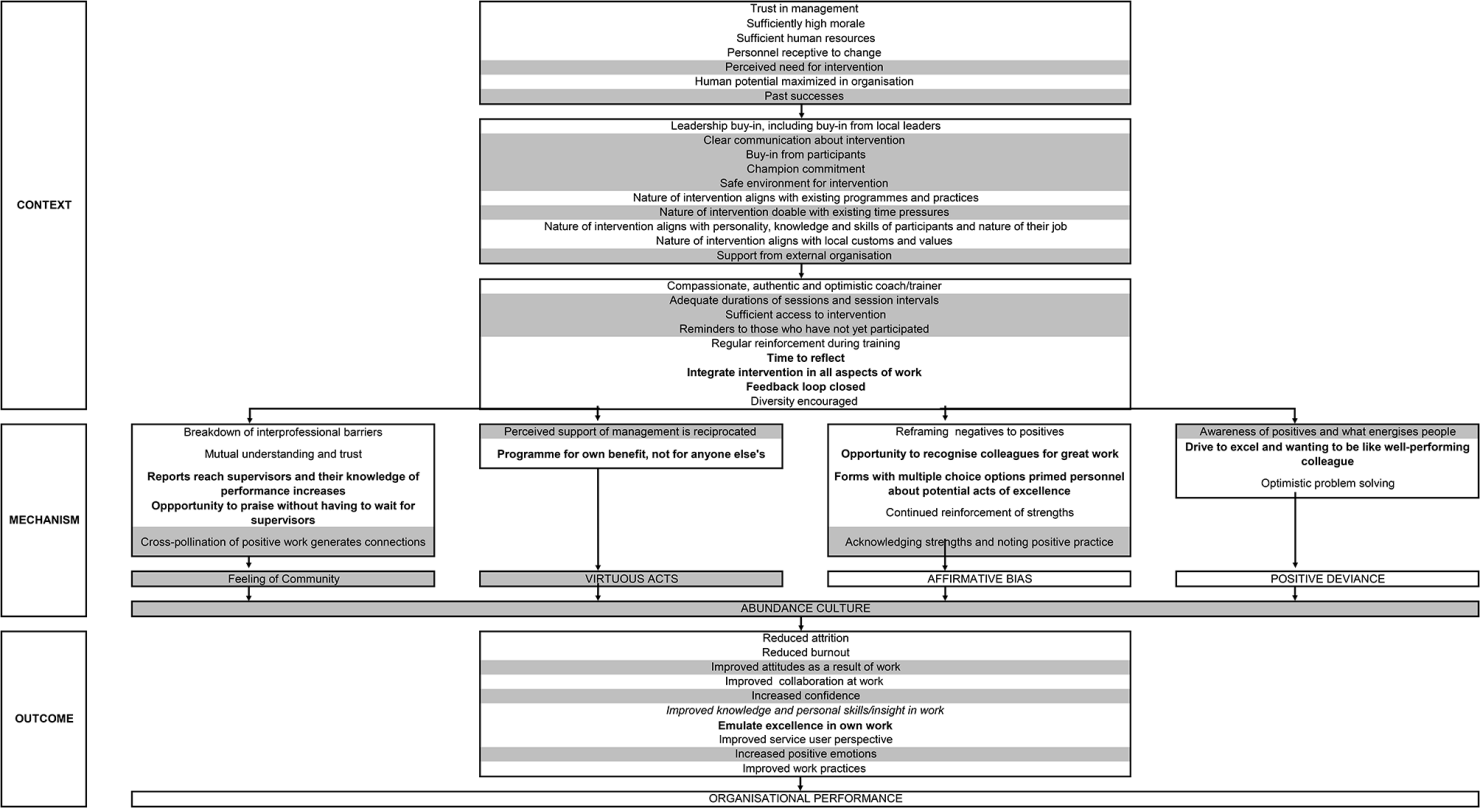
Full adapted logic model after mixed method study

We identified various new factors for the logic model and reinforced factors that have previously been identified. All elements identified in the current study are presented in Fig. 1, while the full updated logic model is shown in Fig. 2. We intended for quantitative and qualitative data to be triangulated with the help of the logic model, but no statistically significant changes were identified in the quantitative data. Additionally, we did not identify perceived supervision, as measured in the questionnaires, as impacted by LfE in the qualitative interviews. While no significant difference was identified in terms of motivation of CHWs in the quantitative findings, CHWs experienced ‘increased positive emotions’, and ‘increased self-esteem’.

## Discussion

Approximately a third of CHWs in Neno District participated in the LfE intervention in the first four months of implementation. Despite lack of statistically significant quantitative findings CHWs welcomed the LfE programme as it made them feel appreciated suggesting there is likely to be some value in an LfE programme. There was variation in participation among the different catchment areas. There are no data available on participation rates in LfE programmes in the UK or elsewhere, making it impossible to compare participation rates.

While the intervention was feasible in Neno District, we identified several barriers and facilitators for implementation. Going forward the LfE forms need further refinement to make the intervention more accessible and valuable to CHWs.

A logic model was developed to explain contextual factors, and mechanisms that could lead to LfE intermediate outcomes for CHWs in Neno District. The developed logic model can be used by those designing and implementing interventions like LfE for health workers.

### Reflection on outcomes of LfE

Stakeholders, CHWs and site supervisors welcomed the LfE intervention for CHWs in Neno District as it allowed them to appreciate CHWs for their excellent work, in the absence of other opportunities to do so. The LfE programme allowed CHWs to identify acts of excellence as they observed other CHWs. Additionally, the forms showed them acts that PIH considered to be excellent. The programme improved stakeholder insights into CHW work on the ground as through completed LfE forms collaborations between CHWs to support clients were identified. CHWs have high workloads and duties at home, the knowledge that someone appreciated them for an act of excellence, by submitting a report, was believed to lead to increased motivation and hard work. While CHWs initially did not believe their work was worthy of praise, LfE allowed them to see their work from a different angle and that it was worthy of praise, which led to increased motivation and hard work. These mechanisms leading to improved motivation were also identified in Ebenso et al.’s evaluation of a maternal and child health programme in Nigeria as ‘fostering peer-support and collegial relations’; ‘making staff feel supported, valued and appreciated’; ‘creating a comfortable working environment’ and ‘boosting morale and confidence’ [39].

While our qualitative findings indicated that motivation could be improved by recognition of CHWs work with the careful introduction of LfE, to meet expectations of participants, the quantitative data did not demonstrate statistically significant improvement in motivation after implementing the co-designed LfE intervention.

We identified that some CHWs expected to be rewarded for their participation in LfE and the lack of financial or in-kind rewards, despite expectations of these, may have led to demotivation. A study by Ormel et al. showed that it is important to provide incentives according to expectations of CHWs, as an expectation gap may lead to demotivation [12]. Communications regarding rewards should be crystal clear going forwards.

We did not identify any impact of LfE on perceived supervision in either the quantitative or the qualitative data, nor was this an outcome that we identified in a systematic review regarding the impact of PP interventions for health workers [24]. The lack of findings in our study could potentially be due to miscommunication as those on the ground believed the intervention was for CHWs only, so those higher up in hierarchy did not participate. While we intended the intervention to be for everyone, as it could lead to an improved feeling of community, CHWs mentioned that they liked that the intervention was aimed at only themselves.

We did however identify the impact of LfE on relationships among CHWs themselves. While celebrating excellence, we also found that the LfE intervention increased jealousy among CHWs and may have emphasises cliques of friends who reported each other. Jealousy was also identified in the exploratory study conducted in the UK [22].

A study regarding the impact of Appreciative Inquiry on healthcare workers in Malawi did not find a statistically significant improvement in job satisfaction after the implementation of the AI intervention, which was potentially due to other factors impacting job satisfaction, including lack of resources [40]. We did not measure job satisfaction alone, but as part of motivation, which did not change after implementation of LfE. However, motivation among CHWs was very high to start with, which could have influenced the lack of improvement.

While we identified similar contextual factors and mechanisms and qualitative outcomes as identified in earlier studies, as of yet there is limited quantitative data available regarding the impact of interventions like LfE on healthcare workers. Future work should further investigate the impact of interventions like LfE on motivation of health workers Additionally, future implementers of LfE should identify how to manage and minimise feelings of jealousy.

### Reflection on implementation and evaluation processes

While we aimed to allow everyone who noticed CHW excellence to report an event, only CHWs participated. It may be that others did not know of the existence of the intervention and did thus not participate or that forms were not easily accessible for those who are based at the district or community hospitals.

We were unsure how well CHWs understood the LfE form and the lack of understanding of the form may have confused CHWs when filling it in and may have prevented them from participating in the programme.

Due to miscommunication, the post-LfE questionnaires did not include Site F names, leading to an underpowered paired analysis. However, the absolute change in medians at Site F was small and it is unlikely that low sample sizes were the reason for no statistically significant difference in motivation or perceived supervision after the implementation of LfE. We were uncertain if CHWs understood the negatively phrased questions in the questionnaire or felt uncomfortable answering them honestly, as the correlation between these questions and the construct of motivation was low. While the Cronbach’s alpha was not very high, we considered it sufficient for a ten-question questionnaire.

The ‘perceived supervision’ outcome, as measured in the questionnaire, was not as relevant as initially expected. Other limitations preventing participation, such as transport challenges and limited boxes may have led to the limited impact of the LfE programme.

Retrospectively, it would have been better to look at other intermediate outcomes, like positive emotion or confidence in work, instead of perceived supervision. This could have been achieved by including CHWs earlier in the research design process. Involving CHWs more in both design of research and development of the LfE form could have led to them understanding the form better.

### Reflections on the logic model

We developed a logic model and identified contextual factors before design and implementation included a perceived need for intervention, previously identified in our systematic review regarding the impact of interventions based on PP for healthcare workers [24]. Past successes seem to impact the uptake of the LfE intervention, as was identified in this study and a previous UK exploratory study [22]. Other identified contextual factors included the cross-pollination of work, as CHWs pause, take time to reflect, and identify the excellence of their colleagues. The feeling of connectedness through professional support structures and decreased distance between CHWs and their supervisors were also identified as necessary for CHW performance, as specified in a comparative analysis of factors shaping CHW relationships in four countries, including Malawi [41].

During the design and implementation, communication about the intervention is particularly important. In our study, many CHWs had difficulties understanding the intervention when it was first introduced, which had been identified previously in our systematic review [24]. ‘Champion commitment’, as specified in the exploratory study [22], the systematic review, and this study seems essential for the intervention uptake [24].

Apart from contextual factors, mechanisms were identified. CHWs wanted their colleagues to feel encouraged, and in return, filling in a report about their excellence made CHWs feel good themselves, which falls under virtuous acts, as identified in previous studies [22, 24]. However, as many CHWs expected rewards, participation in the LfE programme was not necessarily a virtuous act, performed without expecting something in return [42]. LfE provided an opportunity to recognise colleagues for excellence, creating affirmative bias. LfE encouraged CHWs to acknowledge strengths and highlighted positive practices, as previously identified [22, 24]. Through LfE, an awareness of positives and what energises CHWs was created, which was previously identified as necessary for impact. A new factor feeding into positive deviance was the drive to excel and wanting to be like the colleague you are reporting for excellence. LfE could potentially encourage professional support and decrease the distance from supervisors when they participate in the intervention, thus increasing feelings of community and improving performance [41].

LfE is thought to lead to outcomes including improved attitudes to work and improved confidence among health workers, both previously identified as intermediate outcomes. One new output of LfE was identified, namely emulating excellence by those who reported a colleague for excellence. The LfE intervention was believed to improve knowledge and new insight into CHW work practices, which was previously identified in the exploratory study [22]. However, at the time we did not know how this could fit into the logic model. Improved knowledge was not directly identified as impacting the performance of CHWs, although Merriel identified it as a factor that influenced patient outcomes, which are an aspect of performance [40]. Positive emotions, like feeling appreciated, can lead to increased resilience through broadened thought-action-repertoires, as explained by Fredrickson et al. [43] and thus impact the performance of CHWs.

The developed logic model can be used by those designing and implementing interventions like LfE for health workers. While the model was adapted according to our findings of LfE for CHWs, there were similarities between CHWs in the Neno District and employees of hospitals in the UK [22], as well as with similar programmes as identified in a systematic review [24], indicating the logic model could be helpful for health professionals in various settings and increase the strength of implementation of interventions like LfE [44]. The logic model is large and not all the included components may play a role in the impact of LfE. Future studies should test the components of the logic model, preferably with the help of quantitative data as thus far limited quantitative research has been conducted into the impact of LfE or similar programmes on health workers. Testing the different features may provide more insight into their role in LfE outcomes.

### Study strengths and limitations

A strength of this study is that a large number of CHWs and Site supervisors participated in the interviews. While we did not aim for data saturation per se, this seems to have been reached. We purposively selected site supervisors to help explain differences between the sites; these did not appear to be as large as initially anticipated based on the discrepancy in reports provided per site, indicating that site supervisors may have been unaware of their impact on uptake of LfE.

Our study had several limitations, firstly it was conducted in one rural setting and may not be generalisable to other settings, particularly as CHWs in our study receive a stipend, and differ from more professionalised or fully volunteer CHWs. However, the Neno District is comparable to many other rural settings in Sub-Saharan Africa with usefulness and applicability in the findings. Another limitation regarded the COVID-19 pandemic, which impacted CHW workflows, workloads and LfE implementation processes. Remote coordination of implementation activities seems to have led to some omissions, which could have impacted understanding of LfE by CHWs and impacted reporting and types of events reported, as well as expectations regarding rewards. Standardised implementation and more hands-on guidance and training to the Site Supervisors and those implementing LfE, could have improved understanding and consistency among sites (262). Another limitation regarded the limited piloting and co-production activities conducted before roll-out of the intervention, again due to limited time for data collection as implementation got delayed due to the COVID-19 pandemic.

### Recommendations– what now

Success and sustainability of CHW programmes requires ongoing investment in quality training, supervision, mentoring and organisational support (56). The LfE intervention could potentially contribute to this as managers and supervisors are provided with information about what is going well within the CHW programme. In future, other cadres of health workers could be included in the LfE intervention. We did intend for this to happen, but potentially due to miscommunication, this didn’t realise. Involving health professionals from all levels of hierarchy may help CHWs to feel supported and improve their trust in the health system, thereby improving motivation, as explained in previous research (278). Additionally, involving different cadres could help create a feeling of community, which in turn could impact the abundance culture, as explained in the developed logic model.

Due to the importance of community members, including village leaders, in recognising, appreciating and thereby motivating CHWs, in the future, community members could be included in the implementation of LfE. For example, community members could be given the opportunity to report on acts of excellence of CHWs, or feedback on acts of excellence could be provided during village meetings. This could lead to increased perceived support for CHWs, leading to enhanced credibility and community trust.

An exploration into what constitutes excellence from the perspective of various stakeholders involved in LfE could provide those implementing programmes like LfE a better understanding of what excellence entails in the context of the programme and organisation in which LfE will be implemented. The exploration will additionally aid future evaluators of LfE programmes why the programme produced the data it did and may provide a better understanding of if the programme led to desired results.

No randomised controlled trials have been conducted regarding LfE for health workers or CHWs. However, there is a growing body of evidence that suggests interventions like LfE could potentially improve various intermediate outcomes, including motivation of health workers and increased positive emotions, which could lead to improved organisational performance.

A long-term study of impact of LfE, in both the UK and Neno District are important to identify impact once LfE is no longer new. Additionally, long-term data could also inform how the logic model stands up over time.

## Conclusion

LfE allowed for appreciation of excellent work of CHWs, as identified by stakeholders, CHWs, and Site supervisors working in the CHW programme in Neno District, which was believed to lead to improved motivation and hard work. While qualitative findings were positive, we found no statistically significant improvement in motivation or perceived supervision after implementation of LfE. We only measured two potential outcomes of LfE though, and more quantitative data are needed to identify if LfE can make a statistically significant difference in the qualitatively identified outcomes presented in the logic model. Despite high participation, we found that further refinement of the LfE programme and forms through a co-design process with the CHWs could make the programme more accessible and useful for CHWs.

We identified various contextual factors and mechanisms explaining the uptake and outcomes of the LfE intervention and adapted a previously developed logic model accordingly. This logic model can be used by those implementing or evaluating LfE interventions.

### Electronic supplementary material

Below is the link to the electronic supplementary material.

Supplementary Material 1

Supplementary Material 2

Supplementary Material 3

## Data Availability

The quantitative dataset is available from the corresponding author upon request for the purposes of checking our analyses only. Research participants did not consent for their data to be used in future research, so our dataset cannot be used for this. The qualitative dataset generated and analysed during the current study is not publicly available. Even without identifiers such as names, the dataset could potentially hold identifiable participant information in aggregate form due to the catchment area and role within the CHW programme. Neno District is a small district. With potential identifiers, we believe it would be ethically inappropriate to publicly share data that could reveal our participants’ identities if read by someone within the district. The dataset, or part of it, could be available from the corresponding author on reasonable request with permission from the Neno District Research committee.

## Abbreviations

CHW: Community Health Worker
CMO: Context-Mechanism-Outcome
HIV: Human Immunodeficiency Virus
IQR: Interquartile Range
LfE: Learning from Excellence
NHS: National Health Service
PIH: Partners in Health
PP: Positive Psychology
STI: Sexually Transmitted Infections
TB: Tuberculosis
UK: United Kingdom
