# Supplementary material for: Mixed method evaluation of a learning from excellence programme for community health workers in Neno, Malawi
Source: BMC Health Serv Res. 2024 Mar 19;24:355. doi: 10.1186/s12913-024-10686-w (PMC10953074; doi:10.1186/s12913-024-10686-w)
Supplement: Supplementary file 1 — Supplementary Material 1 [file 12913_2024_10686_MOESM1_ESM.docx]

### **Design and implementation of LfE programme in Neno, Malawi.**

### **Background**

#### Co-Design and implementation of the LfE form

LfE is an intervention consisting of three aspects: a form to report excellent events, a feedback loop in which those noted for excellence, as well as potential others (i.e., supervisors or those reporting excellence) are informed about the excellent report, and the follow-up of some excellent reports that stand out to learn more from these events and apply this learning for future improvements.

We followed a design process as described by Eyles et al. (1) for designing mHealth interventions. Various co-design activities, including informal conversations, online feedback, and formal discussions were conducted with key stakeholders in the implementation of the CHW programme in Neno District, including the Chief Medical Officer, the Community Director and the CHW manager and programme officers who all had access to email. Each design activity followed up on results and ideas created in a previous activity (2).

The LfE form that was designed is shown below. The form included multiple choice questions for type of excellent event and an open question where reporters could explain why they thought the event was excellent. The LfE programme was piloted in one catchment area in Neno District, Site A, where it was introduced during the day the CHWs were given their stipends in June 2020. After one month, reports were examined and discussed among members of the CHW team and a researcher. Implementation was adapted according to the outcomes of the discussion. The main adaptation regarded the introduction to the LfE programme as there was some uncertainty if CHWs were reporting excellence or everyday performance. We thus designed some scenarios, one representing everyday performance and one presenting excellence. These scenarios were read out during implementation and a discussion with CHWs was encouraged regarding these different scenarios. During the CHW stipend payday (which brings together CHWs from each catchment area in person) in late August 2020, the co-designed LfE intervention was introduced in all other catchment areas in Neno District.

#### **Methods**

#### Participants

The intervention was open to all CHWs, including senior CHWs in the Neno District, as well as anyone else working at a health facility, who observed a CHW doing something excellent.

#### Data collection

There were 16 LfE reporting boxes in Neno District, one in each of the 14 health facilities and two in remote areas in two catchments for ease of delivery for CHWs. CHWs were encouraged to submit forms during visits to the health facility, especially at monthly meeting times. All forms collected between September 2020 and November 2020 were eligible for analysis. Every month the Site Supervisors would open the LfE boxes in their catchment area, at a time suitable for them. The LfE forms would be sent to the District Health Office (DHO) together with the monthly data performance report of the catchment area in question. At the DHO, the forms were collected by a research assistant who oversaw data collection and scanned the forms and uploaded them to a password-protected shared folder on the University of Warwick server. CHWs were informed they were taking part in a research project and that forms would be analysed, and data stored anonymously. The research assistant also translated the answers provided to the question ‘why was this excellent’.

The following information from the forms and translations was extracted and collated into an Excel file: i) name of the CHW reporting, ii) name of the CHW reported, iii) site where excellent event occurred, iv) type of excellent event and iv) translated explanation of why this excellent event was reported. Once all data were collected, the data file was pseudonymised and names of CHWs replaced with unique codes. An overview of the names with the corresponding codes was kept in a password protected file on a D: drive of the University of Warwick, only accessible by MK.

#### Analysis

##### Quantitative

Descriptive statistics including the percentage of CHWs participating in the LfE intervention in each catchment area were calculated based on CHW workforce numbers per catchment area as per October 2020.

Descriptive analyses were conducted for the following variables: number of submitted reports for each type of excellent event (i.e., counselling on treatment adherence, timely referral to the health facility and encouraging a pregnant woman to attend antenatal care during the first trimester), percentage of CHWs submitting a form per catchment area, how often CHWs were reported for excellence and who reported acts of excellence, i.e., CHW A reports CHW B.

##### Qualitative

The translated explanations as provided in the reports were organised per excellent event they belonged, based on the multiple-choice options on the form to, e.g., ‘made a timely referral’. MK read the translated explanations and took notes regarding initial thoughts while going through the explanations. The notes and explanations were summarised into memos explaining the explanations provided per type of excellent event.

#### **Results**

Of the 555 submitted reports, 123 (22.2%) included multiple excellent events. The percentage of reports containing multiple excellent events varied from 2.9% (n=1) at site K to 50% (n=3) at Site I (table 1).

The percentage of CHWs filling in a report, of the total number of CHWs at the specific site, varied from 5.5% (n=6) in site I to 100% (n=33) in site C (table 2).

In total 162 reports, by 27.7% of CHWs participating in the LfE intervention, involved CHWs reporting the person whom they had been reported by. At some sites this happened more often, for example at site B, 89.9% (n=8) of the participating CHWs reported the same person who they had been reported by, versus 0% of CHWs at site A (n=0) (table 3).

An overview of type of excellent event reported per site is shown in Table 2. Commonly reported excellent events included ‘counselling client on treatment adherence’ and ‘timely referrals’, which consisted of 15.1% (n=84) and 11.2% (n=62) of the excellence reports respectively. On the other hand, 0.9% (n=5) of the reports regarded ‘psychosocial report provided during hospital stay’, which was only reported at 3 sites: site G (0.7%, n=1), site J (5.7%, n=3) and site M (1.8%, n=1).

In total 274 (24.2%) CHW were reported for excellence at least once. Sixty-three CHWs (5.6%) were reported twice, 15 (1.3%) were reported three times, 17 four times (1.5%) and six (0.5%) were reported six times (table 4).

#### Open-ended question explanations

Table 5 provides an overview of typical explanations per type of event, as provided by CHWs who filled in a report. The explanations provided for ‘other’ excellent events, or for forms that included multiple reported excellent events emphasised the hardworking nature of the reported CHW, an explanation of the various excellence events reported on the form or explanation of just one of the reported events on the form. As these were idiosyncratic and varied at an individual level, they are not presented in the table.

Table 5 Typical explanations as provided on the LfE forms as submitted by CHWs between September 2020 and November 2020.

| Excellent event | Typical example |
| --- | --- |
| Advocated well on behalf of the client | Encouraged a client who was reluctant to seek medical attention to go to the hospital for assistance where [client] was diagnosed with [illness] and started receiving treatment. |
| Counselled a client on treatment adherence | Advise and encourages a client to be taking medication every day and as prescribed. |
| Made a timely referral | Saved a life of a patient who was very ill by referring them to the hospital in good time for assistance. |
| Performed three postnatal care visits | Followed up on a woman who had just given birth, for three consecutive times. |
| Provided psychosocial support to client during admission and/or hospital stay | Managed to give encouragements to a client who was admitted at the hospital. |
| Referred a vulnerable household to POSER/other relevant services | Connected a certain family in [CHWs'] village with POSER department to receive assistance. |
| Regularly refer suspected malnutrition cases | Excelled on the part of referring a child whom CHW was suspecting to be malnourished to the hospital where the child was put on special diet. |
| Submit client’s sputum on a regular basis | [CHW] is excellent when it comes to frequent sputum collection and sending to the hospital for analysis. |
| Supported a client to attend family planning services | Encouraged a woman to start using family planning methods. |
| Supported a defaulting patient to go back into care | Encouraged a client who had stopped taking medication in the right way to start taking the drugs by following the right prescription. |
| Supported a pregnant woman to go for an antenatal care visit in the first trimester | Encouraged an expectant mother to start antenatal care in her first trimester. |

CHW = Community Health Worker

POSER = Programme on Social and Economic Rights

For forms containing multiple excellent events, there were three types of explanations: 1.) Explanations mentioned the hardworking nature of the CHW, 2.) One of the checked events was explained, 3.) All (or more than one) of the checked events was explained. We did not notice any differences in explanations provided among sites.

The explanations as provided for the ‘advocated well on behalf of patient’ differed from what we had anticipated in design of the form, namely CHWs advocating on behalf of a patient when visiting the health facility. Instead, most explanations focused on ‘encouraging a patient to attend a hospital for treatment or to stick to the treatment regime as prescribed’. Explanations as provided for ‘counselling a patient on treatment adherence’ as well as ‘supporting a defaulting patient back into care’ also regarded CHWs encouraging a patient to attend the hospital for treatment or to stick to treatment regime as prescribed.

Some explanations seemed to regard a different event from the one ticked in the boxes. It may be that the CHWs checked the wrong box or wanted to add some additional information about the excellent performance of the CHW in question.

**Discussion**

Additionally, we did not intend for multiple events to be reported on one form, but this happened often. Various excellent events, including ‘advocating on behalf of the client’, ‘counselling on treatment adherence’ and ‘supporting a defaulting patient back into care’, all included similar explanations by CHWs, indicating that CHWs may not be able to distinguish between these types of excellent events. It could be considered to merge ‘supporting a defaulting patient into care’ and ‘counselling a client on treatment adherence’ into one type of excellent event. The ‘advocating on behalf of a client’ event should be further explained or adjusted in a collaborative process with CHWs, so the excellent event is fully understood, acceptable for CHWs, and can be consistently recorded. Despite the co-design of the LfE form with Community Health leadership in the district, we recognise that CHWs themselves are important stakeholders. Involving CHWs more in the co-design programme could potentially have prevented a lack of understanding. In future work, training and additional support should be provided to the Site supervisors and those implementing the programme.

**References**

1. Eyles H, Jull A, Dobson R, Firestone R, Whittaker R, Te Morenga L, et al. Co-design of mHealth Delivered Interventions: A Systematic Review to Assess Key Methods and Processes. . Current Nutrition Reports. 2016;5:160-7.

2. Clemensen J, Rothmann M, Smith A, Caffery L, Danbjorg D. Participatory design methods in telemedicine research. . Journal of Telemedicine and Telecare. 2016.

**Table 1 Overview of type of excellent event report, in alphabetical order, n (% within site)**

| Type of excellent event | Site | | | | | | | | | | | | | |  |
| --- | --- | --- | --- | --- | --- | --- | --- | --- | --- | --- | --- | --- | --- | --- | --- |
|  | **A** | **B** | **C** | **D** | **E** | **F** | **G** | **H** | **I** | **J** | **K** | **L** | **M** | **Total** | |
| Advocated for client | 0  (0%) | 1  (7.7%) | 5  (8.1%) | 1  (3.2%) | 3  (6.4%) | 2  (5.7%) | 17 (11.6%) | 1  (7.7%) | 0  (0%) | 5  (9.4%) | 1  (2.9%) | 4  (9.5%) | 4  (7.0%) | **44 (7.9%)** | |
| Counselled on treatment adherence | 2  (14.3) | 1  (7.7%) | 7 (11.3%) | 8 (25.8%) | 11 (23.4%) | 5 (14.3%) | 24 (16.3%) | 0  (0%) | 0  (0%) | 5  (9.4%) | 6 (17.1%) | 3  (7.1%) | 12 (21.1%) | **84 (15.1%)** | |
| Timely referral to facility | 3  (21.4%) | 2 (15.4%) | 2  (3.2%) | 3  (9.7%) | 5 (10.6%) | 5 (14.3%) | 17 (11.6%) | 0  (0%) | 3  (50%) | 5  (9.4%) | 3  (8.6%) | 2  (4.8%) | 12 (21.1%) | **62 (11.2%)** | |
| Performed recommended three PNC visits | 0  (0%) | 1  (7.7%) | 3  (4.8%) | 1  (3.2%) | 3  (6.4%) | 1  (2.9%) | 18 (12.3%) | 0  (0%) | 0  (0%) | 6 (11.3%) | 3  (8.6%) | 2  (4.8%) | 3  (5.3%) | **41 (7.4%)** | |
| Psychosocial support during hospital stay | 0  (0%) | 0  (0%) | 0  (0%) | 0  (0%) | 0  (0%) | 0  (0%) | 1  (0.7%) | 0  (0%) | 0  (0%) | 3  (5.7%) | 0  (0%) | 0  (0%) | 1  (1.8%) | **5**  **(0.9%)** | |
| Referred household to POSER | 1  (7.1%) | 1  (7.7%) | 3  (4.8%) | 1  (3.2%) | 2  (4.3%) | 2  (5.7%) | 4  (2.7%) | 1  (7.7%) | 0  (0%) | 1  (1.9%) | 1  (2.9%) | 0  (0%) | 4  (7.0%) | **21**  **(3.8%)** | |
| Referred suspected malnutrition cases | 0  (0%) | 0  (0%) | 5  (8.1%) | 0  (0%) | 1  (2.1%) | 1  (2.9%) | 3  (2.0%) | 0  (0%) | 0  (0%) | 0  (0%) | 2  (5.7%) | 1  (2.4%) | 3  (5.3%) | **16 (2.9%)** | |
| Regular TB sputum submission | 0  (0%) | 0  (0%) | 3  (4.8%) | 0  (0%) | 2  (2.1%) | 3  (8.6%) | 5  (3.4%) | 1  (7.7%) | 0  (0%) | 0  (0%) | 7 (20.0%) | 4  (9.5%) | 3  (5.3%) | **28**  **(5.1%)** | |
| Supported attendance of FP | 2  (14.3%) | 1  (7.7%) | 4  (6.5%) | 3  (9.7%) | 4  (8.5%) | 2  (5.7%) | 11 (7.5%) | 1  (7.7%) | 0  (0%) | 5  (9.4%) | 1  (2.9%) | 2  (4.8%) | 0  (0%) | **36 (6.5%)** | |
| Supported patient to go back into care | 1  (7.1%) | 1  (7.7%) | 4  (6.5%) | 1  (3.2%) | 1  (2.1%) | 2  (5.7%) | 8  (5.4%) | 1  (7.7%) | 0  (0%) | 16 (30.2%) | 1  (2.9%) | 3  (7.1%) | 5  (8.8%) | **44 (7.9%)** | |
| Supported client for ANC visit in the first trimester | 3 (21.4%) | 3 (23.1%) | 1  (1.6%) | 0  (0%) | 4  (8.5%) | 1  (2.9%) | 10 (6.8%) | 1  (7.7%) | 0  (0%) | 2  (3.8%) | 4 (11.4%) | 3  (7.1%) | 1  (1.8%) | **33 (6.0%)** | |
| Checked multiple | 2  (14.3%) | 2 (15.4%) | 25 (40.3%) | 12 (38.7%) | 11 (23.4%) | 8 (22.9%) | 26 (17.7%) | 4 (30.8%) | 3  (50%) | 3  (5.7%) | 1  (2.9%) | 18 (42.9%) | 8 (14.0%) | **123 (22.2%)** | |
| Other, please specify | 0  (0%) | 0  (0%) | 0  (0%) | 1  (3.2%) | 0  (0%) | 3  (8.6%) | 3  (2.0%) | 3 (23.1%) | 0  (0%) | 2  (3.8%) | 5 (14.3%) | 0  (0%) | 1  (1.8%) | **18 (3.2%)** | |
| Total | **14** | **13** | **62** | **31** | **47** | **35** | **147** | **13** | **6** | **53** | **35** | **42** | **57** | **555** | |

ANC = Antenatal Care

FP = Family Planning

PNC = Postnatal care

POSER = Programme on Social and Economic Rights

| Site | Number of CHWs submitting a report (n) | Number of reports submitted by CHW (n) | Percentage of CHW submitting report per total number of CHWs (%) | Total number of CHWs (n)^a^ |
| --- | --- | --- | --- | --- |
| A | 14 | 14 | 70.0% | 20 |
| B | 9 | 13 | 17.7% | 51 |
| C | 33 | 62 | 100% | 33 |
| D | 31 | 31 | 29.8% | 104 |
| E | 43 | 43 | 37.4% | 115 |
| F | 25 | 30 | 18.9% | 132 |
| G | 61 | 131 | 63.5% | 96 |
| H | 11 | 12 | 20.0% | 55 |
| I | 6 | 6 | 5.5% | 110 |
| J | 33 | 49 | 33.4% | 98 |
| K | 29 | 34 | 29.9% | 97 |
| L | 41 | 41 | 44.1% | 93 |
| M | 54 | 55 | 41.9% | 129 |
| Total | **390** | **521** | **34.4%** | **1133** |

**Table 2 Overview of CHWs reporting excellent events between September 2020 and November 2020.**

a. As per 22-10-2020.

a. As per 22-10-2020.

CHW = Community Health Worker

| Site | Number of reports involving CHWs reporting each other (n) | Number of CHWs involved in reporting each other | Percentage of CHWs participating in LfE reporting each other (%) | Total number of CHWs participating^a^ |
| --- | --- | --- | --- | --- |
| A | 0 | 0 | 0% | 14 |
| B | 8 | 8 | 89.9% | 9 |
| C | 21 | 15 | 45.5% | 33 |
| D | 8 | 8 | 25.8% | 31 |
| E | 10 | 10 | 23.3% | 43 |
| F | 16 | 14 | 64.0% | 25 |
| G | 66 | 36 | 59.0% | 61 |
| H | 2 | 2 | 18.2% | 11 |
| I | 2 | 2 | 33.3% | 6 |
| J | 17 | 11 | 33.3% | 33 |
| K | 2 | 2 | 6.9% | 29 |
| L | 6 | 6 | 14.7% | 41 |
| M | 4 | 4 | 7.4% | 54 |
| Total | **162** | **108** | **27.7%** | **390** |

Table 3 CHWs reporting each other for excellence between September 2020 and November 2020.

a. As per 22-10-2020.

CHW = Community Health Worker

Table 5 Number of excellent event reports per CHW reported between September 2020 and November 2020.

| Site | CHWs reported once | CHWs reported twice | CHW reported three times | CHWs reported four times | CHWs reported five times |
| --- | --- | --- | --- | --- | --- |
| A | 12 | 1 |  |  |  |
| B | 8 | 1 | 1 |  |  |
| C | 10 | 18 | 4 | 1 |  |
| D | 30 |  |  |  |  |
| E | 39 | 4 |  |  |  |
| F | 32 |  | 1 |  |  |
| G | 29 | 11 | 6 | 14 | 3 |
| H | 11 | 1 |  |  |  |
| I | 6 |  |  |  |  |
| J | 19 | 10 |  | 2 | 1 |
| K | 17 | 4 | 1 |  | 1 |
| L | 27 | 5 |  |  | 1 |
| M | 34 | 8 | 2 |  |  |
| Total | **274** | **63** | **15** | **17** | **6** |

CHW = Community Health Worker

**Final LfE form**

| **Your Name** |  |
| --- | --- |

| **Are you** | |
| --- | --- |
|  | Community Health Worker |
|  | Leadership |
|  | Senior Community Health Worker |
|  | Site Supervisor |
|  | Health Facility Staff |
|  | Clinical Staff |
|  | Other, please specify: |

| **Name of person who achieved excellence** |  |
| --- | --- |

| **What catchment area do they work in** | | | |
| --- | --- | --- | --- |
|  | Site A |  | Site H |
|  | Site B |  | Site I |
|  | Site C |  | Site J |
|  | Site D |  | Site K |
|  | Site E |  | Site L |
|  | Site F |  | Site M |
|  | Site G |  | Site N |

| **What did they do that was excellent?** | |
| --- | --- |
|  | Advocated well on behalf of the client |
|  | Counselled a client on treatment adherence |
|  | Made a timely referral |
|  | Performed three post-natal care visits |
|  | Provided psychosocial support to client during admission and/or hospital stay |
|  | Referred a vulnerable household to POSER/other relevant services |
|  | Regularly refer suspected malnutrition cases |
|  | Submit client’s sputum on a regular basis |
|  | Supported a client to attend family planning services |
|  | Supported a defaulting patient to go back into care |
|  | Supported a pregnant woman to go for an antenatal care visit in the first trimester |
|  | Other, please specify: |

| **Why was this excellent** |
| --- |
|  |
