# Supplementary figures and images for: Mixed method evaluation of a learning from excellence programme for community health workers in Neno, Malawi
Source: BMC Health Serv Res. 2024 Mar 19;24:355. doi: 10.1186/s12913-024-10686-w (PMC10953074; doi:10.1186/s12913-024-10686-w)

**Supplementary file 2: Logic Model Starting Point**


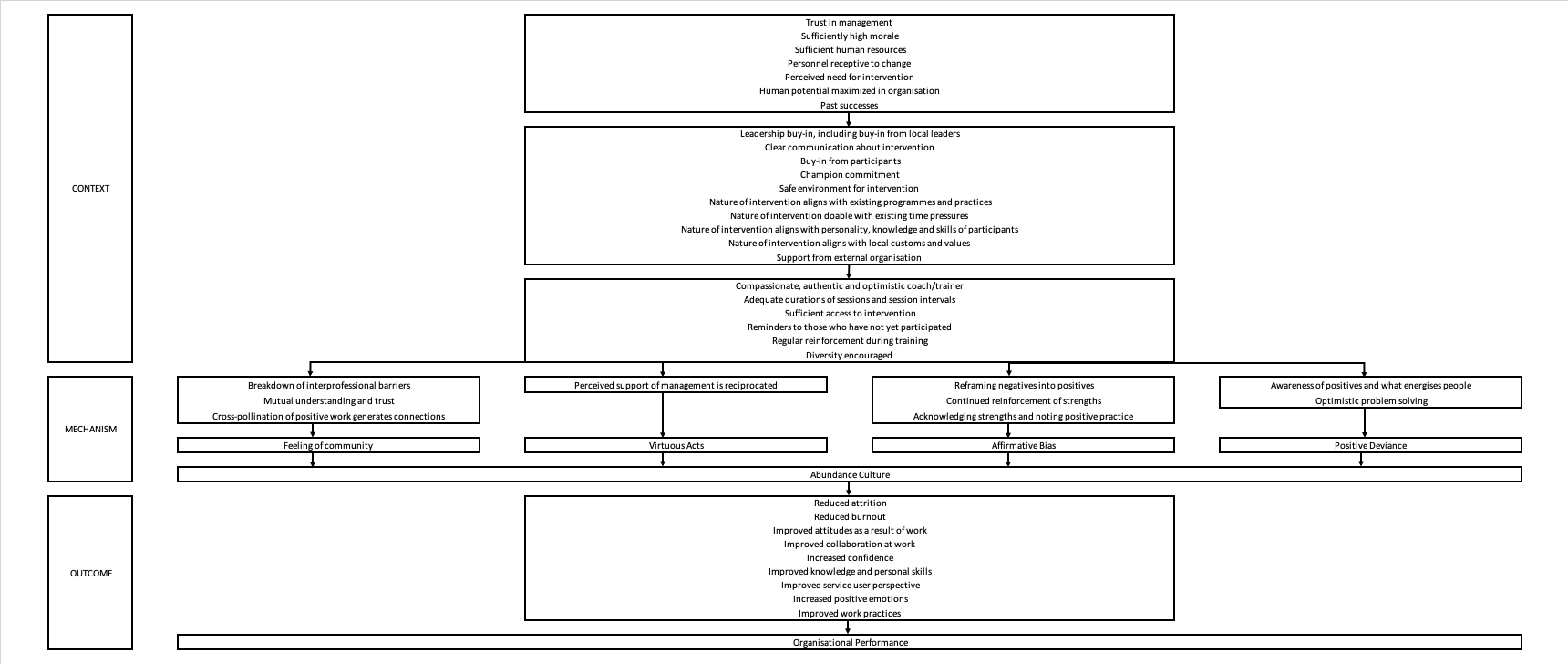

Supplement: Supplementary file 2 — Supplementary Material 2 [file 12913_2024_10686_MOESM2_ESM.docx]
