# Supplementary material for: Mixed method evaluation of a learning from excellence programme for community health workers in Neno, Malawi
Source: BMC Health Serv Res. 2024 Mar 19;24:355. doi: 10.1186/s12913-024-10686-w (PMC10953074; doi:10.1186/s12913-024-10686-w)
Supplement: Supplementary file 3 — Supplementary Material 3 [file 12913_2024_10686_MOESM3_ESM.docx]

**Supplementary file 4**

Cronbach α of questionnaire if question is removed.

| Concept | Question | Cronbach α | |
| --- | --- | --- | --- |
|  |  | Before | After |
| Motivation | Q1 In general, I am satisfied with this job | 0.54 | 0.44 |
|  | Q2 If I could, I would do a different job | 0.60 | 0.55 |
|  | Q3 I feel my work is appreciated and valued by the SCHW and Site Supervisor | 0.51 | 0.49 |
|  | Q4 I am proud to be a CHW | 0.55 | 0.43 |
|  | Q5 I feel part of the Community Health Team | 0.57 | 0.44 |
|  | Q7 I feel motivated to work as hard as I can | 0.56 | 0.38 |
|  | Q8 I only do this job so I get paid at the end of the month | 0.65 | 0.59 |
| Perceived Supervision | Q6 I feel able to discuss work-related problems with other CHWs and SCHW | 0.55 | 0.59 |
|  | Q9 The SCHW meets with me regularly | 0.52 | 0.44 |
|  | Q10 The SCHW helps me to update my knowledge and skills | 0.55 | 0.44 |
| Total |  | 0.58 | 0.50 |

CHW = Community Health Worker

SCHW = Senior Community Health Worker

**Spearman correlations between individual question responses questionnaire before implementation of Learning from Excellence, n=43.**

|  |  | Q1 | Q2 | Q3 | Q4 | Q5 | Q7 | Q8 | Q6 | Q9 | Total Motivation | Total Supervision | Total |
| --- | --- | --- | --- | --- | --- | --- | --- | --- | --- | --- | --- | --- | --- |
| Motivation | Q1 In general, I am satisfied with this job | **-** |  |  |  |  |  |  |  |  |  |  |  |
|  | Q2 If I could, I would do a different job | 0.25 | - |  |  |  |  |  |  |  |  |  |  |
|  | Q3 I feel my work is appreciated and valued by the SCHW and Site Supervisor | 0.63 | 0.26 | - |  |  |  |  |  |  |  |  |  |
|  | Q4 I am proud to be a CHW | 0.43 | 0.17 | 0.47 | - |  |  |  |  |  |  |  |  |
|  | Q5 I feel part of the Community Health Team | 0.14 | 0.14 | 0.31 | 0.30 | - |  |  |  |  |  |  |  |
|  | Q7 I feel motivated to work as hard as I can | 0.43 | 0.19 | 0.39 | 0.53 | 0.11 | - |  |  |  |  |  |  |
|  | Q8 I only do this job so I get paid at the end of the month | -0.11 | -0.13 | -0.09 | -0.21 | 0.12 | 0.11 | - |  |  |  |  |  |
| Perceived Supervision | Q6 I feel able to discuss work-related problems with other CHWs and SCHW | 0.23 | -0.14 | 0.26 | 0.12 | 0.26 | 0.12 | -0.04 | - |  |  |  |  |
|  | Q9 The SCHW meets with me regularly | 0.33 | -0.07 | 0.28 | 0.37 | 0.17 | 0.35 | 0.19 | 0.37 | - |  |  |  |
|  | Q10 The SCHW helps me to update my knowledge and skills | 0.32 | -0.06 | 0.27 | 0.03 | 0.19 | 0.33 | 0.26 | 0.03 | 0.13 |  |  |  |
|  |  |  |  |  |  |  |  |  |  |  | **0.21** | **0.18** | **0.19** |

CHW = Community Health Worker SCHW = Senior Community Health Worker

Spearman correlations between individual question responses questionnaire after implementation of Learning from Excellence, n=35.

|  |  | Q1 | Q2 | Q3 | Q4 | Q5 | Q7 | Q8 | Q6 | Q9 | Mean Motivation | Mean Supervision | Mean |
| --- | --- | --- | --- | --- | --- | --- | --- | --- | --- | --- | --- | --- | --- |
| Motivation | Q1 In general, I am satisfied with this job | - |  |  |  |  |  |  |  |  |  |  |  |
|  | Q2 If I could, I would do a different job | 0.36 | - |  |  |  |  |  |  |  |  |  |  |
|  | Q3 I feel my work is appreciated and valued by the SCHW and Site Supervisor | -0.03 | -0.19 | - |  |  |  |  |  |  |  |  |  |
|  | Q4 I am proud to be a CHW | 0.55 | 0.26 | -0.26 | - |  |  |  |  |  |  |  |  |
|  | Q5 I feel part of the Community Health Team | 0.31 | 0.15 | 0.18 | 0.28 | - |  |  |  |  |  |  |  |
|  | Q7 I feel motivated to work as hard as I can | 0.37 | 0.09 | -0.02 | 0.36 | 0.41 | - |  |  |  |  |  |  |
|  | Q8 I only do this job so I get paid at the end of the month | -0.11 | -0.13 | 0.06 | -0.21 | -0.07 | -0.05 | - |  |  |  |  |  |
| Perceived Supervision | Q6 I feel able to discuss work-related problems with other CHWs and SCHW | 0.02 | -0.19 | 0.01 | 0.26 | 0.10 | 0.22 | 0.02 | - |  |  |  |  |
|  | Q9 The SCHW meets with me regularly | 0.25 | 0.00 | -0.09 | 0.25 | 0.00 | 0.11 | 0.00 | 0.30 | - |  |  |  |
|  | Q10 The SCHW helps me to update my knowledge and skills | 0.16 | 0.22 | -0.06 | 0.25 | 0.20 | 0.36 | -0.01 | -0.01 | 0.10 |  |  |  |
|  |  |  |  |  |  |  |  |  |  |  | **0.11** | **0.13** | **0.11** |

CHW = Community Health Worker SCHW = Senior Community Health Worker

**Median and interpolated median scores for 10-item questionnaire, by site and total overall, before implementation of Learning from Excellence.**

|  |  | Site F | | | Site G | | | All | | |
| --- | --- | --- | --- | --- | --- | --- | --- | --- | --- | --- |
|  |  | Median (IQR) | IM | N | Median (IQR) | IM | N | Median (IQR) | IM | N |
| Motivation | Q1 In general, I am satisfied with this job | 5  (5 to 5) | 4.88 | 25 | 4  (4 to 5) | 4.28 | 25 | 5  (4 to 5) | 4.64 | 50 |
|  | Q2 If I could, I would do a different job | 2  (1 to 4) | 1.93 | 24 | 1  (1 to 2) | 1.30 | 24 | 1.5  (1 to 2) | NA | 48 |
|  | Q3 I feel my work is appreciated and valued by the SCHW and Site Supervisor | 5  (5 to 5) | 4.89 | 23 | 5  (4 to 5) | 4.54 | 25 | 5  (4 to 5) | 4.75 | 48 |
|  | Q4 I am proud to be a CHW | 5  (4 to 5) | 4.81 | 25 | 5  (4 to 5) | 4.72 | 25 | 5  (4 to 5) | 4.76 | 50 |
|  | Q5 I feel part of the Community Health Team | 5  (4 to 5) | 4.81 | 25 | 5  (4 to 5) | 4.54 | 25 | 5  (4 to 5) | 4.69 | 50 |
|  | Q7 I feel motivated to work as hard as I can | 5  (4 to 5) | 4.76 | 25 | 4  (4 to 5) | 4.38 | 25 | 5  (4 to 5) | 4.70 | 50 |
|  | Q8 I only do this job so I get paid at the end of the month | 4  (3 to 5) | 4.08 | 21 | 5  (5 to 5) | 4.70 | 24 | 5  (4 to 5) | 4.52 | 45 |
| Perceived Supervision | Q6 I feel able to discuss work-related problems with other CHWs and SCHW | 5  (4 to 5) | 4.79 | 24 | 5  (4 to 5) | 4.76 | 25 | 5  (4 to 5) | 4.78 | 49 |
|  | Q9 The SCHW meets with me regularly | 5  (4 to 5) | 4.72 | 25 | 5  (4 to 5) | 4.67 | 25 | 5  (4 to 5) | 4.69 | 50 |
|  | Q10 The SCHW helps me to update my knowledge and skills | 5  (5 to 5) | 4.95 | 24 | 5  (5 to 5) | 4.88 | 25 | 5  (5 to 5) | 4.92 | 49 |
| Total (maximum score is 50) |  | 45  (41 to 47) | 44.75 | 19 | 41.5  (40 to 45) | NA | 24 | 43  (41 to 46) | 43.00 | 43 |

**Median and interpolated median scores for10-item questionnaire, by site and total overall, after implementation of Learning from Excellence**

|  |  | Site F | | | Site G | | | All | | |
| --- | --- | --- | --- | --- | --- | --- | --- | --- | --- | --- |
|  |  | Median (IQR) | IM | N | Median (IQR) | IM | N | Median (IQR) | IM | N |
| Motivation | Q1 In general, I am satisfied with this job | 5  (4 to 5) | 4.77 | 22 | 4  (4 to 5) | 4.33 | 23 | 5  (4 to 5) | 4.62 | 44 |
|  | Q2 If I could, I would do a different job | 2  (1 to 3) | 1.30 | 22 | 1  (1 to 2) | 1.42 | 22 | 2  (1 to 3) | 1.82 | 43 |
|  | Q3 I feel my work is appreciated and valued by the SCHW and Site Supervisor | 5  (4 to 5) | 4.70 | 24 | 5  (4 to 5) | 4.68 | 23 | 5  (4 to 5) | 4.69 | 47 |
|  | Q4 I am proud to be a CHW | 5  (4 to 5) | 4.77 | 22 | 5  (4 to 5) | 4.71 | 22 | 5  (4 to 5) | 4.73 | 43 |
|  | Q5 I feel part of the Community Health Team | 5  (5 to 5) | 4.85 | 22 | 5  (4 to 5) | 4.77 | 22 | 5  (4 to 5) | 4.82 | 42 |
|  | Q7 I feel motivated to work as hard as I can | 5  (4 to 5) | 4.82 | 23 | 4  (4 to 5) | 4.73 | 23 | 5  (4 to 5) | 4.80 | 45 |
|  | Q8 I only do this job so I get paid at the end of the month | 5  (3 to 5) | 3.61 | 23 | 5  (5 to 5) | 4.14 | 23 | 4  (4 to 5) | 4.09 | 46 |
| Perceived Supervision | Q6 I feel able to discuss work-related problems with other CHWs and SCHW | 5  (4 to 5) | 4.82 | 23 | 5  (4 to 5) | 4.78 | 23 | 5  (4 to 5) | 4.80 | 46 |
|  | Q9 The SCHW meets with me regularly | 5  (4 to 5) | 4.81 | 22 | 5  (4 to 5) | 4.62 | 23 | 5  (4 to 5) | 4.72 | 45 |
|  | Q10 The SCHW helps me to update my knowledge and skills | 5  (5 to 5) | 4.95 | 23 | 5  (5 to 5) | 4.82 | 23 | 5  (5 to 5) | 4.89 | 46 |
| Total (maximum score is 50) |  | 45  (43 to 46) | 45.00 | 18 | 42  (41 to 45) | 42.10 | 20 | 44  (42 to 46) | 44.00 | 35 |
